# PopulationProfiler: A Tool for Population Analysis and Visualization of Image-Based Cell Screening Data

**DOI:** 10.1371/journal.pone.0151554

**Published:** 2016-03-17

**Authors:** Damian J. Matuszewski, Carolina Wählby, Jordi Carreras Puigvert, Ida-Maria Sintorn

**Affiliations:** 1 Science for Life Laboratory, Uppsala, Sweden; 2 Science for Life Laboratory, Stockholm, Sweden; 3 Centre for Image Analysis, Department of Information Technology, Uppsala University, Uppsala, Sweden; 4 Division of Translational Medicine and Chemical Biology, Department of Medical Biochemistry and Biophysics, Karolinska Institutet, Stockholm, Sweden; Texas A&M University, UNITED STATES

## Abstract

Image-based screening typically produces quantitative measurements of cell appearance. Large-scale screens involving tens of thousands of images, each containing hundreds of cells described by hundreds of measurements, result in overwhelming amounts of data. Reducing per-cell measurements to the averages across the image(s) for each treatment leads to loss of potentially valuable information on population variability. We present PopulationProfiler—a new software tool that reduces per-cell measurements to population statistics. The software imports measurements from a simple text file, visualizes population distributions in a compact and comprehensive way, and can create gates for subpopulation classes based on control samples. We validate the tool by showing how PopulationProfiler can be used to analyze the effect of drugs that disturb the cell cycle, and compare the results to those obtained with flow cytometry.

## Introduction

Automated image-based high-content microscopy provides a platform for phenotypic screening of complex compound libraries and drug combination sets [1]. Image processing and analysis tools enable automated extraction of large numbers of quantitative measurements describing the phenotype on a single cell basis [2]. Predicting and characterizing the mechanism of action of each compound in a large library typically requires careful analysis of this multidimensional data. However, many studies reduce per-cell measurements to population means, leading to loss of potentially valuable information about population heterogeneity [3, 4]. Such an approach is not very surprising considering the complexity of handling hundreds of measurements from hundreds of cells per treatment, in assays often spanning libraries of thousands of compound-dose combinations.

There are commercially available software that allow definition and quantification of subpopulations such as Screener by GeneData, SpotFire by TIBCO, IN Cell Investigator Software by GE Healthcare, and Harmony by PerkinElmer. Additionally, the machine-learning tools within CellProfiler Analyst [5] and other software [6] can be trained to identify and count cells belonging to different sub-populations. However, to our knowledge, no simple, free and open source tools for full-plate visualization of per-cell measurement distributions has previously been presented.

We present PopulationProfiler, software that allows visualization of histograms and sub-population distribution of high-content screening data stored in the common csv text file format. The main idea is to reduce per-cell measurements to per-well distributions, each represented by a histogram, and optionally further reduce the histograms to sub-type counts based on gating (setting bin ranges) of known control distributions and local adjustments to histogram shape. Such analysis is necessary in a wide variety of applications, e.g. DNA damage assessment using foci intensity distributions, assessment of cell type specific markers, and cell cycle analysis. We show how PopulationProfiler can be used for cell cycle perturbation, protein translocation, and EdU incorporation analysis.

PopulationProfiler is written in Python which makes it platform independent. The source code, sample dataset and an executable program (for Windows only) are freely available at http://cb.uu.se/~damian/PopulationProfiler.html.

## Methodology

**Fig 1:**
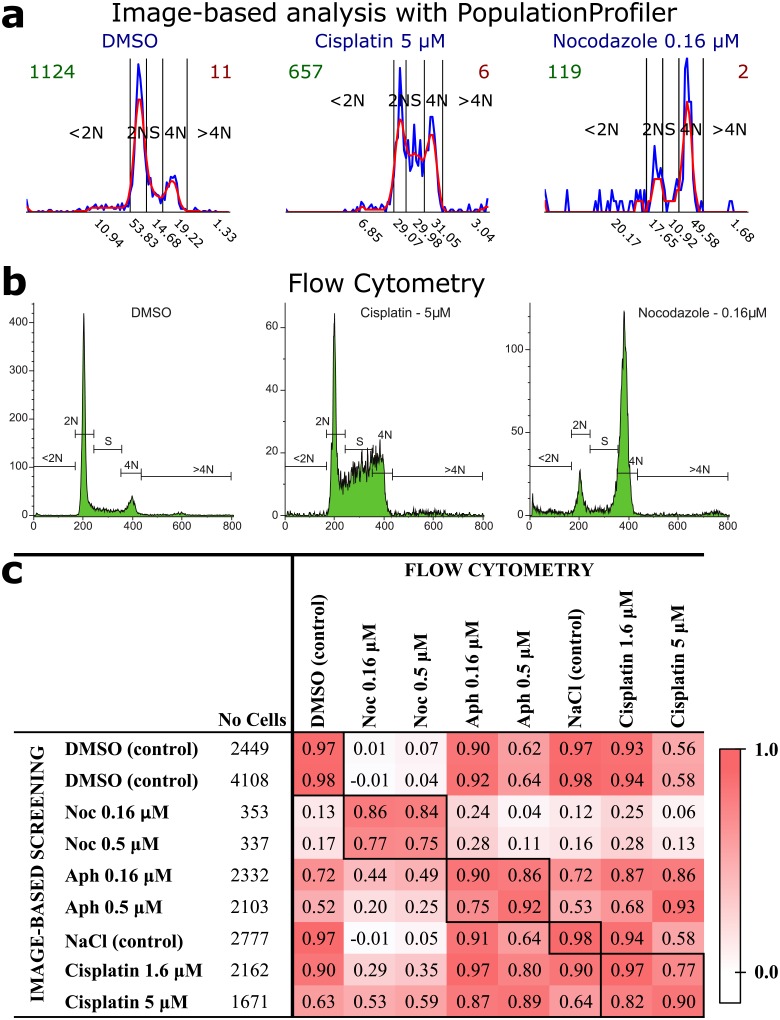
Image-based cell cycle analysis of cell line A549 with PopulationProfiler and its comparison to flow cytometry. a) DNA content histograms created with PopulationProfiler. The blue and red lines show data before and after smoothing, respectively. The numbers under the x-axis present the percentage contribution of each cell cycle sub-population. b) The corresponding cell cycle analysis with flow cytometry. c) A comparison of the results (the contributions of the 5 cell cycle sub-populations) reveals high correlation. The respective total cell counts used by PopulationProfiler and flow cytometry are 18292 and 102751.

PopulationProfiler’s simple graphical user interface (GUI) imports data from image-based screening measurements; it allows selection of multiple csv files containing information on treatment and position (well) within a multi-well plate. Each file is considered as an independent experiment with rows representing individual cell measurements. One type of measurement is processed at a time and cells are grouped (aggregated) based on well labels. The labels for cell aggregation and the measurement are selected by the user from a drop-down list created from the csv file header (first row). The GUI also allows selection of control wells based on the treatment labels (there can be more than one well per treatment). If such labels are not available, the user can select control wells manually. The corresponding data is pooled and stored as a separate record in the output csv file. PopulationProfiler thereafter calculates and displays the distribution of the selected measurement as a histogram for each well (Fig 1a). A vector representation of each well’s histogram is saved in the output file, and can be used as input for e.g., cluster analysis, elsewhere. The cell count for each well is also saved as a measure of statistical relevance of population effects. A very low cell count usually indicates cell death, and morphological measurements are then less likely to convey useful information.

### Case study—cell cycle analysis

A commonly studied treatment response is disruption of the cell cycle. We therefore added functionality specialized for analysis of relative per-cell DNA content, measured as log2 of the integrated intensity of a DNA stain such as DAPI, Hoechst or PI [7]. For an unperturbed cell population, a histogram of the DNA content typically consists of two peaks, as shown in Fig 1a (DMSO). The higher peak to the left (2N) corresponds to the larger part of the cell population with a single copy of the genome, whereas the smaller peak on the right (4N), corresponds to the sub-population that has doubled the amount of DNA. Before exploring the effect of treatments that potentially perturb the cell cycle, PopulationProfiler allows the user to set bin ranges (subpopulation gates) using data from untreated control wells. Values corresponding to the centers of the 2N and 4N sub-populations are defined as the largest and second largest maximum respectively, and all DNA intensity measurements are normalized such that the maximum of the 2N peak corresponds to 1 and the center of the 4N peak corresponds to 2. In order to avoid multiple peaks at 2N and 4N locations the histograms are smoothed with a Gaussian filter (*σ* = 1.5). Individual cells are thereafter assigned to five classes named <2N, 2N, S, 4N, and >4N based on thresholds at 0.75, 1.25, 1.75 and 2.5 respectively, in accordance with [7]. During analysis of treated wells, thresholds are automatically adjusted to the shape of each well’s histogram within limits defined by the 2N and 4N peaks of the untreated wells. This adaptive gating allows a comparison of cell cycle effects decoupled from changes in, e.g., cell size or uptake of DNA stain. The alternative, i.e. using the gates found for the negative controls for all the other samples, is also possible with the PopulationProfiler. In addition, the tool allows setting manual customized gates which gives more analysis possibilities (arbitrary number and range of non-overlapping gates) to the user.

## Results

The experiments performed had two goals; to compare population data collected by the presented image-based cell cycle analysis approach using PopulationProfiler to population data collected by flow cytometry (one dimensional intensity measurements), and to compare their ability to detect treatments that disturb the cell population. We tested the two cell cycle analysis approaches on a cancer cell line (lung cancer, A549, known to be sensitive to cell cycle perturbants) and a slow replicating control cell line (insensitive to cell-cycle perturbations non-transformed colon epithelial, CCD841) exposed to five treatments (DMSO, Aphidicolin, Nocodazole, NaCl and Cisplatin) at one or two doses, as detailed in the supplementary material. Cell cycle histograms were compared visually (Fig 1a and 1b and Figs B and C in S1 Text), and by calculating Pearson’s correlation coefficient of normalized cell cycle sub-population distribution vectors found using PopulationProfiler and Beckman Coulter Kaluza software for the flow cytometry data. Data from the drug sensitive cell line (A549) showed high Pearson’s correlation coefficient for all equal drug-dose comparisons (Fig 1c and Fig D in S1 Text) and relatively high correlation for different doses of the same drug, while noticeably lower correlation between the effect of different drugs. A very similar pattern appears when comparing replicates of the flow cytometry experiments, while no effects were observed for the more stable CCD841 cell line (Fig E in S1 Text). More results, detailed experiment description and discussion can be found in S1 Text, together with example of protein translocation and EdU incorporation analysis.

## Discussion

Rather than reducing per-cell measurements to population averages, PopulationProfiler allows data reduction while maintaining information on population heterogeneity. We show that PopulationProfiler keeps enough information to discriminate between drugs that perturb the cell cycle with similar detail as obtained by flow cytometry, but at significantly lower cell counts. Image based analysis allows efficient discrimination between true signals and artifacts and PopulationProfiler enables comparison of measurements of morphological features, such as sub-cellular signal localization and cytoskeletal patterns, not possible to observe by flow cytometry.

## Supporting Information

S1 Text PopulationProfiler: Supplementary Material. This file contains the user manual, additional application examples, and the detailed description and discussion of the presented cell cycle analysis comparison.
(PDF)
