# Supplementary material for: PopulationProfiler: A Tool for Population Analysis and Visualization of Image-Based Cell Screening Data
Source: PLoS One. 2016 Mar 17;11(3):e0151554. doi: 10.1371/journal.pone.0151554 (PMC4795740; doi:10.1371/journal.pone.0151554)
Supplement: S1 Text — This file contains the user manual, additional application examples, and the detailed description and discussion of the presented cell cycle analysis comparison. (PDF) [file pone.0151554.s001.pdf]

# PopulationProfiler: a tool for population analysis and visualization of image-based cell screening data. Supplementary Material

Damian J. Matuszewski[1,3,*], Carolina Wählby[1,3], Jordi Carreras Puigvert[2,4,‡], and Ida-Maria Sintorn[1,3,‡]

[1]Science for Life Laboratory, Uppsala, Sweden,

[2]Science for Life Laboratory, Stockholm, Sweden,

[3]Centre for Image Analysis, Uppsala University, Uppsala, Sweden,

[4]Helleday Laboratory, Division of Translational Medicine and Chemical Biology, Karolinska Institutet, Stockholm, Sweden.

[‡]These authors contributed equally to this work.

*corresponding author: damian.matuszewski@it.uu.se

## 1 APPLICATION - TRANSLOCATION ASSAY

Reducing per-cell measurements to the population statistics finds applications in many studies. In order to demonstrate this we used image set BBBC013v1 provided by Ilya Ravkin, available from the Broad Bioimage Benchmark Collection (Ljosa et al. 2012). It consists of a translocation assay of the Forkhead (FKHR-EGFP) fusion protein from the cytoplasm to the nucleus in stably transfected human osteosarcoma cells, U2OS. CellProfiler (Carpenter et al., 2006) was used to segment the cell nuclei in the images by first Gaussian smoothing with sigma of 1 and then by the Maximum Correlation Thresholding method (Padmanabhan et al. 2010) on the whole image followed by exclusion of small objects. No background correction was applied. The cytoplasm was defined as a small neighborhood surrounding each nucleus. The correlation between the GFP and DNA stains was then computed in the cell area. The doses of the two drugs used for building the dataset (Wortmannin and LY294002) were chosen so that the arrest of the Forkhead protein in the nuclei is observed at medium drug dose. Figure A presents selected wells from the dataset. It is clearly visible that the correlation between GFP and DNA signals increases with Wortmannin dose.

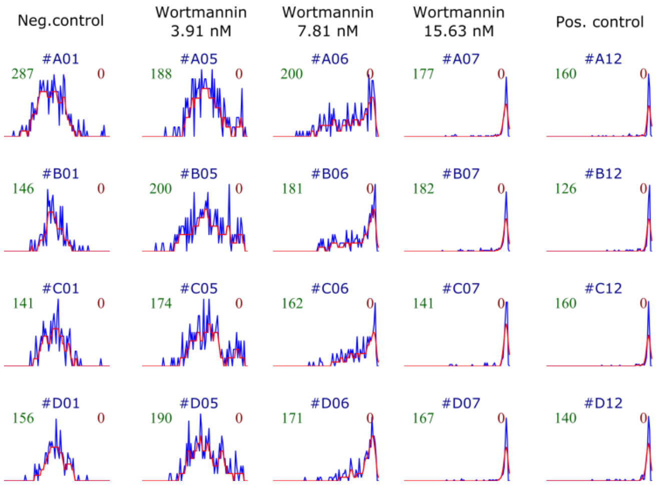


**Figure A.** Correlation between GFP and DNA stains histograms generated by PopulationProfiler. Each graph shows data from one well. The blue plot is the actual data, whereas the red is the Gaussian ($\sigma = 1.5$) smoothed curve. The green number in the top-left corner is the total cell count in the well and the red number in the top-right corner is the sum of cell counts in extreme bins of the histogram.

# 2 APPLICATION - CELL CYCLE ANALYSIS EXPERIMENT

We used two different cell lines (lung cancer A549 and colon epithelial non-transformed CCD841) exposed to 5 different treatments: Dimethyl Sulfoxide (DMSO), Aphidicolin, Nocodazole, NaCl and Cisplatin. DMSO and NaCl are commonly used reagents to dissolve compounds or drugs; hence, we used them as negative controls. The remaining treatments are known to affect the cell cycle:

- Aphidicolin: inhibits DNA synthesis by restraining DNA polymerase alpha and delta (blocks the cell cycle at early S phase);
- Nocodazole: inhibits microtubule polymerization (arrests cells in a 4N or >4N phase);
- Cisplatin: is an intercalating drug that creates intrastrand crosslinks in the DNA , which ultimately triggers apoptosis (programmed cell death).

The cell lines were obtained from ATCC and maintained in Dulbecco's Modified Eagle Medium (Invitrogen) supplemented with 10% fetal bovine serum (Invitrogen) and 1% penicillin/streptomycin (Invitrogen), at 37ºC and 5% CO2. NaCl and Cisplatin formulated in 0.9% NaCl were purchased from Hospira; Aphidicolin and Nocodazole were purchased from Sigma-Aldrich and dissolved in DMSO from Merck.

## 2.1 Experimental setup

### *1.1.1 Image-based screening (IBS)*

A549 and CCD841 cells were seeded in an imaging 384 well plate (Falcon) 24h prior to exposure to the compounds at a density of 1000 and 2500 cells per well, respectively. The cells were then exposed to the vehicle (DMSO or NaCl), 0.16µM-0.5µM of Aphidicolin, 0.16µM-0.5µM of Nocodazole, and 1.6µM-5µM of Cisplatin for 24h. The cells were then fixed in 4% paraformaldehyde (PFA) in PBS (Santa Cruz) for 15 minutes, and 2µg/ml Hoechst 33342 (Sigma-Aldrich) in Phosphate Buffered Saline (PBS) (Invitrogen) was added for 15 minutes to stain the DNA. Three wells were used for each drug-dose combination and the negative controls (DMSO and NaCl). Subsequently, the cells were imaged with an ImageXpress (Molecular Devices) high-throughput microscope. At this point, the sample preparation has typically taken approximately 3h and 30 minutes, with minimal volumes used given the microwell plate format. Next, CellProfiler (Carpenter et al., 2006) was used to segment the cell nuclei by Gaussian smoothing followed by watershed segmentation combined with Otsu thresholding and exclusion of small objects. No background correction was applied. Finally, the total DNA content (integrated intensity of the DNA stain) was measured per nucleus. These measurements were then analyzed with PopulationProfiler. The software analyzed pooled histograms from the negative control wells for each cell line to determine the integrated intensity values corresponding to the centers of the 2N and 4N sub-populations. These values were then applied as input parameters to define a search range for the exact 2N and 4N DNA peaks for each well and to normalize DNA intensity, such that the maximum of the 2N peak corresponds to 1 and the center of the 4N DNA peak corresponds to 2. Individual cells were thereafter categorized automatically to one of the following five sub-populations according to DNA content:

- < 2N – all cells with DNA intensity below 0.75,
- 2N – DNA intensity between 0.75 and 1.25,
- S – DNA intensity between 1.25 and 1.75,
- 4N – DNA intensity between 1.75 and 2.5,
- > 4N – DNA intensity above 2.5 (Chan et al., 2013).

In order to avoid multiple peaks at 2N and 4N locations the histograms were smoothed with a Gaussian filter ($\sigma = 1.5$).

### *1.1.2 Flow cytometry (FC)*

A549 and CCD841 cells were seeded in 24 well plates (Greiner) at a density of 50.000 and 75.000 cells per well, respectively. After 24h, the cells were exposed to the corresponding concentrations of the aforementioned compounds for 24h. Two wells were used for each drug-dose combination and the negative controls. Next, the cells were trypsinized, and collected into 1.5ml Eppendorf tubes to be pelleted by centrifugation and washed once with PBS. Subsequently the cells were lysed in Vindelöv's PI solution containing propidium iodide (PI), Tris, NaCl, Tergitol-type NP-40 and RNase (all from Sigma-Aldrich). The cells were then incubated for 1h at 4ºC in the dark, to allow for the staining of the DNA, and subsequently analyzed by flow cytometry using a Beckman Coulter Navios. At this point, the sample preparation and analysis has typically taken approximately 5 to 6h. In the case of a flow cytometer capable of analyzing samples in 96 well plate format this time may be shorter. The analysis of the data was done with the Beckman Coulter Kaluza software. It is to be noted that the described procedure is intended to maintain the nuclei intact but there is a large loss of cells mainly due to the trypsinization and washing steps. Upon initial data acquisition of the samples, a size exclusion (gating) was applied to ensure single cell population measurements by excluding cell debris and cell doublets. Next, the corresponding different cell cycle phase gates were set for the negative control (DMSO) as a reference and left unaltered for the rest of the samples.

## 2.2 Results

Figure B shows DNA intensity histograms generated with PopulationProfiler. Each graph shows data from one representative well for each drug-dose combination. The vertical black lines mark the automatically adjusted divisions into the 5 cell cycle subpopulations. Figure C presents the corresponding histograms obtained with the flow cytometer (FC).

Figure D-A presents Pearson's correlation coefficients between normalized cell cycle subpopulation distribution vectors found with IBS + PopulationProfiler and FC for the A549 cell line. Figures D-B and D-C present similar calculations but in this case comparing results within PopulationProfiler and FC respectively. For the print clarity Fig. D present pooled data from multiple runs of the same experiments – there were two replicates for each drug-dose combination in FC and three in IBS. Similar results were obtained when individual experiment runs were compered to one another.

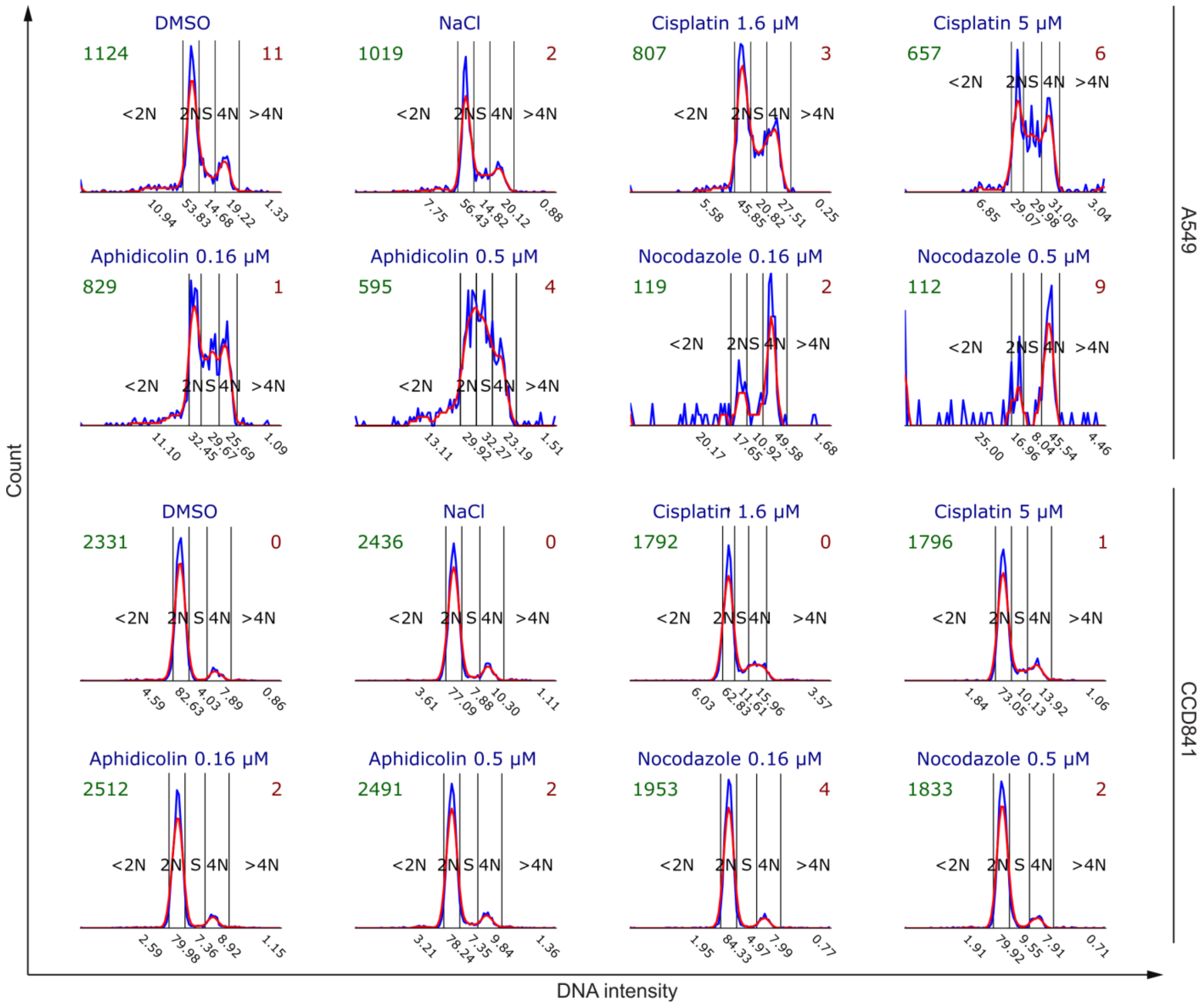


**Figure B.** DNA intensity histograms generated by PopulationProfiler. Each graph shows data from a representative well for each of the drug-dose combinations. The blue plot is the actual data, whereas the red is the smoothed curve with Gaussian ($\sigma = 1.5$) used for finding the subpopulation bins (marked with vertical black lines). The green number in the top-left corner is the total cell count in the well and the red number in the top-right corner is the sum of cell counts in extreme bins of the histogram. The numbers under x-axis are the percentages of cells in each of the 5 cell cycle subpopulations.

In all three tables of Figure D the background color is scaled for each value so that white corresponds to high correlation and dark red to low. The characteristic "cross" pattern (corresponding to high drug response to Nocodazole – a drug affecting cell cycle by arresting cells in the 4N phase) is visible in all three tables, which shows that both approaches provide similar results and can be successfully used for cell cycle analysis.

The cell count in case of IBS was much lower than in FC (see Fig. D). Nevertheless, even in the least populous case of Nocodazole it was still sufficient to observe clear drug response. This indicates that much less cells are needed in case of image-based screening to perform cell cycle analysis. Especially considering the large initial amount of cells required (and lost) during the FC sample preparation protocols (trypsinization, spinning, and washing).

Comparison of the results from the presented image-based DNA content analysis with those obtained using flow cytometry shows high correlation between the two approaches. The Pearson's correlation coefficient for corresponding results is above 75 % for all tested drug-dose combinations and above 90% in more than 66 % of cases. The lowest correlation is observed for the two doses of Nocodazole (86 % and 75 % for 1.6 and 5 µM respectively). The reasons for that are partly very low number of cells in the IBS analysis (these are the two least populated samples), and partly the fact that Nocodazole has a strong effect on the cell cycle and as the gating is not exactly the same in the two approaches the effect

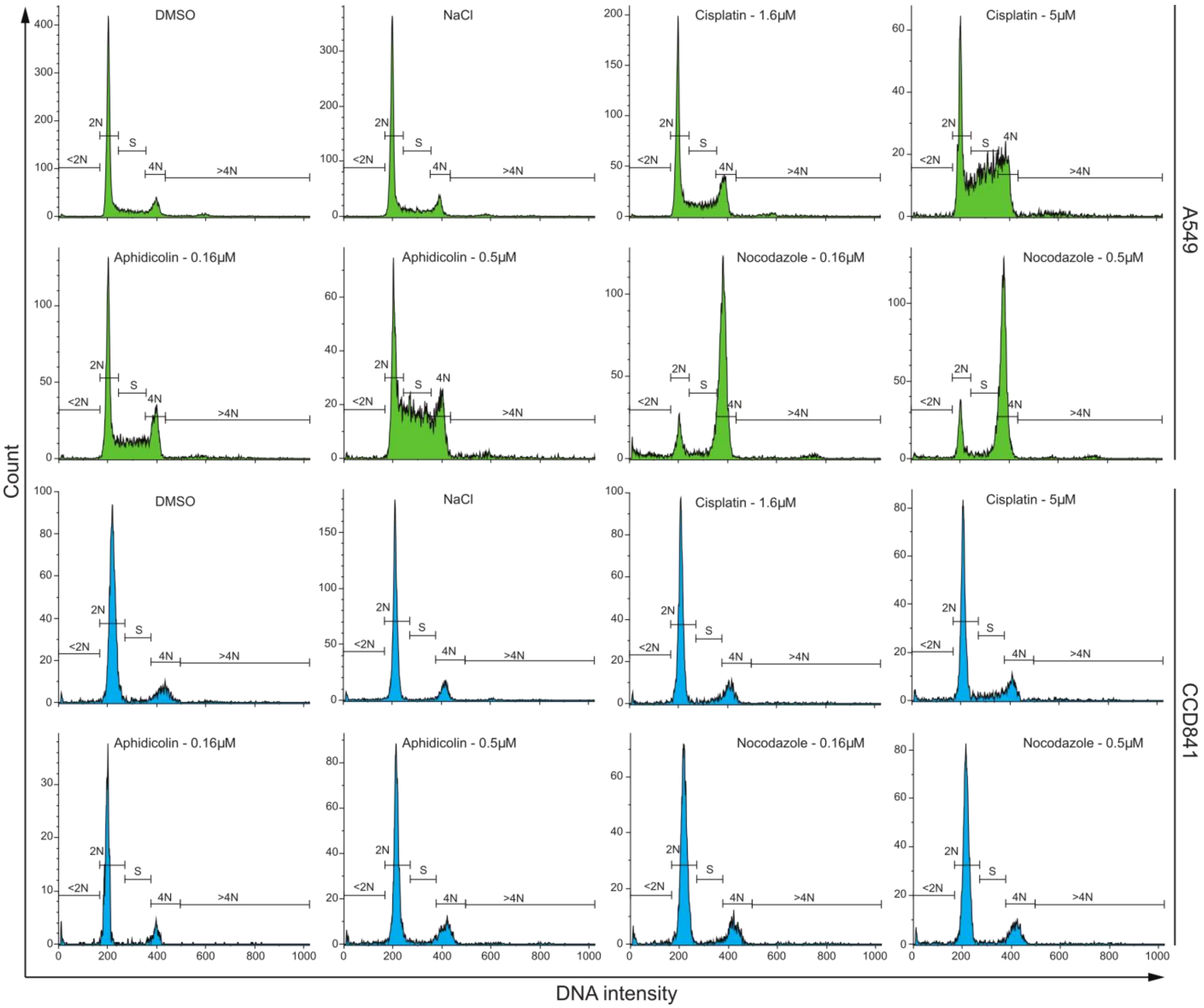


**Figure C.** DNA content histograms obtained with the flow cytometer. Each graph shows data from a representative well for each of the drug-dose combinations. The division into cell cycle subpopulations (horizontal bars in the graphs) was set manually for the negative controls and used for all drug-dose combinations in the corresponding cell line.

manifests in slightly different ways (some cells classified as 4N in FC were considered as > 4N by PopulationProfiler).

Figure E corresponds to Fig. D but for the CCD841 cell line data. CCD841 being non-cancer cells divide slower than the cancer cells from A549. Therefore, they have much more time to repair the damage caused by the drugs that target specifically the cell cycle, and hence, are also less vulnerable to these drugs. Therefore, the CCD841 cell cycle profile should not change as much as the one of the cancer cells, and thus was used as the negative control for the experiment. This cell line showed no response in the DNA content to any of the drugs. Therefore, all the histograms presented in Figures B and C from the cell line CCD841 look similar which resulted in flat high correlation coefficients in Fig. E.

In order to better compare the amount of cells necessary to perform the cell cycle analysis we randomly subsampled the acquired FC data. For each sample 150000 records, which corresponds to approximately half of the total, were randomly selected. The results are presented in Fig. F. The histograms and subpopulations look similar to the initial ones, however, the definition, and therefore, the correct classification of the cells in their corresponding phase becomes more erratic as the number of cells decreases. It can be also easily observed that only a small fraction of measurements captured with the flow cytometry are actually individual cells suitable for analysis (the rest being debris and clumped cells). Moreover, it is crucial to note that the majority of cells are lost during the sample preparation and data acquisition processes. This made us conclude that in practice flow cytometry uses the biological samples much less efficiently, and hence, requires more cells to perform the analysis.

**A**

| IMAGE-BASED SCREENING | No Cells | FLOW CYTOMETRY: DMSO (control) | Noc 0.16 µM | Noc 0.5 µM | Aph 0.16 µM | Aph 0.5 µM | NaCl (control) | Cisplatin 1.6 µM | Cisplatin 5 µM |
|---|---|---|---|---|---|---|---|---|---|
| **DMSO (control)** | 2449 | 0.97 | 0.01 | 0.07 | 0.90 | 0.62 | 0.97 | 0.93 | 0.56 |
| **DMSO (control)** | 4108 | 0.98 | -0.01 | 0.04 | 0.92 | 0.64 | 0.98 | 0.94 | 0.58 |
| **Noc 0.16 µM** | 353 | 0.13 | 0.86 | 0.84 | 0.24 | 0.04 | 0.12 | 0.25 | 0.06 |
| **Noc 0.5 µM** | 337 | 0.17 | 0.77 | 0.75 | 0.28 | 0.11 | 0.16 | 0.28 | 0.13 |
| **Aph 0.16 µM** | 2332 | 0.72 | 0.44 | 0.49 | 0.90 | 0.86 | 0.72 | 0.87 | 0.86 |
| **Aph 0.5 µM** | 2103 | 0.52 | 0.20 | 0.25 | 0.75 | 0.92 | 0.53 | 0.68 | 0.93 |
| **NaCl (control)** | 2777 | 0.97 | -0.01 | 0.05 | 0.91 | 0.64 | 0.98 | 0.94 | 0.58 |
| **Cisplatin 1.6 µM** | 2162 | 0.90 | 0.29 | 0.35 | 0.97 | 0.80 | 0.90 | 0.97 | 0.77 |
| **Cisplatin 5 µM** | 1671 | 0.63 | 0.53 | 0.59 | 0.87 | 0.89 | 0.64 | 0.82 | 0.90 |

**B**

| IMAGE-BASED SCREENING | IMAGE-BASED SCREENING: DMSO (control) | DMSO (control) | Noc 0.16 µM | Noc 0.5 µM | Aph 0.16 µM | Aph 0.5 µM | NaCl (control) | Cisplatin 1.6 µM | Cisplatin 5 µM |
|---|---|---|---|---|---|---|---|---|---|
| **DMSO (control)** | 1.00 | 1.00 | 0.28 | 0.35 | 0.79 | 0.59 | 1.00 | 0.93 | 0.68 |
| **DMSO (control)** | 1.00 | 1.00 | 0.23 | 0.31 | 0.78 | 0.60 | 1.00 | 0.93 | 0.68 |
| **Noc 0.16 µM** | 0.28 | 0.23 | 1.00 | 0.98 | 0.49 | 0.20 | 0.25 | 0.42 | 0.49 |
| **Noc 0.5 µM** | 0.35 | 0.31 | 0.98 | 1.00 | 0.57 | 0.33 | 0.32 | 0.48 | 0.54 |
| **Aph 0.16 µM** | 0.79 | 0.78 | 0.49 | 0.57 | 1.00 | 0.89 | 0.79 | 0.95 | 0.98 |
| **Aph 0.5 µM** | 0.59 | 0.60 | 0.20 | 0.33 | 0.89 | 1.00 | 0.60 | 0.77 | 0.89 |
| **NaCl (control)** | 1.00 | 1.00 | 0.25 | 0.32 | 0.79 | 0.60 | 1.00 | 0.93 | 0.68 |
| **Cisplatin 1.6 µM** | 0.93 | 0.93 | 0.42 | 0.48 | 0.95 | 0.77 | 0.93 | 1.00 | 0.90 |
| **Cisplatin 5 µM** | 0.68 | 0.68 | 0.49 | 0.54 | 0.98 | 0.89 | 0.68 | 0.90 | 1.00 |

**C**

| FLOW CYTOMETRY | No Cells | DMSO (control) | Noc 0.16 µM | Noc 0.5 µM | Aph 0.16 µM | Aph 0.5 µM | NaCl (control) | Cisplatin 1.6 µM | Cisplatin 5 µM |
|---|---|---|---|---|---|---|---|---|---|
| **DMSO (control)** | 19142 | 1.00 | -0.05 | 0.01 | 0.93 | 0.65 | 1.00 | 0.96 | 0.58 |
| **Noc 0.16 µM** | 11784 | -0.05 | 1.00 | 1.00 | 0.20 | 0.17 | -0.06 | 0.16 | 0.21 |
| **Noc 0.5 µM** | 10518 | 0.01 | 1.00 | 1.00 | 0.27 | 0.25 | 0.00 | 0.23 | 0.29 |
| **Aph 0.16 µM** | 9997 | 0.93 | 0.20 | 0.27 | 1.00 | 0.86 | 0.93 | 0.99 | 0.82 |
| **Aph 0.5 µM** | 10519 | 0.65 | 0.17 | 0.25 | 0.86 | 1.00 | 0.65 | 0.81 | 1.00 |
| **NaCl (control)** | 17480 | 1.00 | -0.06 | 0.00 | 0.93 | 0.65 | 1.00 | 0.96 | 0.59 |
| **Cisplatin 1.6 µM** | 14115 | 0.96 | 0.16 | 0.23 | 0.99 | 0.81 | 0.96 | 1.00 | 0.76 |
| **Cisplatin 5 µM** | 9196 | 0.58 | 0.21 | 0.29 | 0.82 | 1.00 | 0.59 | 0.76 | 1.00 |

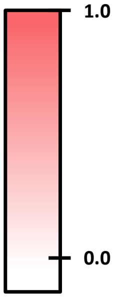


**Figure D.** Pearsson's correlation coefficients of normalized cell cycle subpopulation vectors – image-based screening vs. flow cytometry (A), image-based screening vs. image-based screening (B), and flow cytometry vs. flow cytometry (C). Various treatments: Aphidicolin (Aph), Nocodazole (Noc), NaCl and Cisplatin were applied to cell line A549. The drug dose is stated by the name (in µM). Dark background indicates high correlation.

**A**

| IMAGE-BASED SCREENING | No Cells | FLOW CYTOMETRY: DMSO (control) | Noc 0.16 µM | Noc 0.5 µM | Aph 0.16 µM | Aph 0.5 µM | NaCl (control) | Cisplatin 1.6 µM | Cisplatin 5 µM |
|---|---|---|---|---|---|---|---|---|---|
| **DMSO (control)** | 7489 | 1.00 | 1.00 | 0.99 | 1.00 | 0.99 | 1.00 | 0.99 | 0.99 |
| **DMSO (control)** | 7577 | 1.00 | 1.00 | 0.99 | 1.00 | 0.99 | 1.00 | 0.99 | 0.99 |
| **Noc 0.16 µM** | 5907 | 1.00 | 0.99 | 0.99 | 1.00 | 0.99 | 1.00 | 0.99 | 0.99 |
| **Noc 0.5 µM** | 5763 | 0.99 | 0.99 | 0.99 | 1.00 | 0.99 | 1.00 | 0.99 | 0.99 |
| **Aph 0.16 µM** | 7446 | 0.99 | 0.99 | 0.99 | 1.00 | 0.99 | 1.00 | 0.99 | 0.99 |
| **Aph 0.5 µM** | 7429 | 0.99 | 0.99 | 0.99 | 1.00 | 0.98 | 0.99 | 0.99 | 0.99 |
| **NaCl (control)** | 7020 | 1.00 | 0.99 | 0.99 | 1.00 | 0.99 | 1.00 | 0.99 | 0.99 |
| **Cisplatin 1.6 µM** | 5399 | 0.99 | 0.99 | 0.99 | 0.99 | 0.99 | 0.99 | 0.99 | 1.00 |
| **Cisplatin 5 µM** | 4455 | 0.99 | 0.99 | 0.99 | 0.99 | 0.99 | 0.99 | 0.99 | 1.00 |

**B**

| IMAGE-BASED SCREENING | No Cells | IMAGE-BASED SCREENING: DMSO (control) | DMSO (control) | Noc 0.16 µM | Noc 0.5 µM | Aph 0.16 µM | Aph 0.5 µM | NaCl (control) | Cisplatin 1.6 µM | Cisplatin 5 µM |
|---|---|---|---|---|---|---|---|---|---|---|
| **DMSO (control)** | 7489 | 1.00 | 1.00 | 1.00 | 1.00 | 1.00 | 1.00 | 1.00 | 1.00 | 0.99 |
| **DMSO (control)** | 7577 | 1.00 | 1.00 | 1.00 | 1.00 | 1.00 | 1.00 | 1.00 | 1.00 | 0.99 |
| **Noc 0.16 µM** | 5907 | 1.00 | 1.00 | 1.00 | 1.00 | 1.00 | 1.00 | 1.00 | 1.00 | 0.99 |
| **Noc 0.5 µM** | 5763 | 1.00 | 1.00 | 1.00 | 1.00 | 1.00 | 1.00 | 1.00 | 0.99 | 0.99 |
| **Aph 0.16 µM** | 7446 | 1.00 | 1.00 | 1.00 | 1.00 | 1.00 | 1.00 | 1.00 | 0.99 | 0.99 |
| **Aph 0.5 µM** | 7429 | 1.00 | 1.00 | 1.00 | 1.00 | 1.00 | 1.00 | 1.00 | 0.99 | 0.99 |
| **NaCl (control)** | 7020 | 1.00 | 1.00 | 1.00 | 1.00 | 1.00 | 1.00 | 1.00 | 1.00 | 0.99 |
| **Cisplatin 1.6 µM** | 5399 | 1.00 | 1.00 | 1.00 | 0.99 | 0.99 | 0.99 | 1.00 | 1.00 | 1.00 |
| **Cisplatin 5 µM** | 4455 | 0.99 | 0.99 | 0.99 | 0.99 | 0.99 | 0.99 | 0.99 | 1.00 | 1.00 |

**C**

| FLOW CYTOMETRY | No Cells | FLOW CYTOMETRY: DMSO (control) | Noc 0.16 µM | Noc 0.5 µM | Aph 0.16 µM | Aph 0.5 µM | NaCl (control) | Cisplatin 1.6 µM | Cisplatin 5 µM |
|---|---|---|---|---|---|---|---|---|---|
| **DMSO (control)** | 6299 | 1.00 | 1.00 | 1.00 | 1.00 | 1.00 | 1.00 | 1.00 | 1.00 |
| **Noc 0.16 µM** | 5613 | 1.00 | 1.00 | 1.00 | 1.00 | 1.00 | 1.00 | 1.00 | 1.00 |
| **Noc 0.5 µM** | 6321 | 1.00 | 1.00 | 1.00 | 0.99 | 1.00 | 1.00 | 1.00 | 1.00 |
| **Aph 0.16 µM** | 1538 | 1.00 | 1.00 | 0.99 | 1.00 | 0.99 | 1.00 | 0.99 | 0.99 |
| **Aph 0.5 µM** | 5483 | 1.00 | 1.00 | 1.00 | 0.99 | 1.00 | 1.00 | 1.00 | 1.00 |
| **NaCl (control)** | 7162 | 1.00 | 1.00 | 1.00 | 1.00 | 1.00 | 1.00 | 1.00 | 1.00 |
| **Cisplatin 1.6 µM** | 5855 | 1.00 | 1.00 | 1.00 | 0.99 | 1.00 | 1.00 | 1.00 | 1.00 |
| **Cisplatin 5 µM** | 5328 | 1.00 | 1.00 | 1.00 | 0.99 | 1.00 | 1.00 | 1.00 | 1.00 |

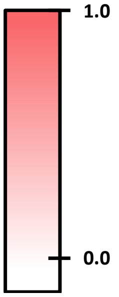


**Figure E.** Pearsson's correlation coefficients of normalized cell cycle subpopulation vectors – image-based screening vs. flow cytometry (A), image-based screening vs. image-based screening (B), and flow cytometry vs. flow cytometry (C). Various treatments: Aphidicolin (Aph), Nocodazole (Noc), NaCl and Cisplatin were applied to cell line CCD841. The drug dose is stated by the name (in µM). Dark background indicates high correlation.

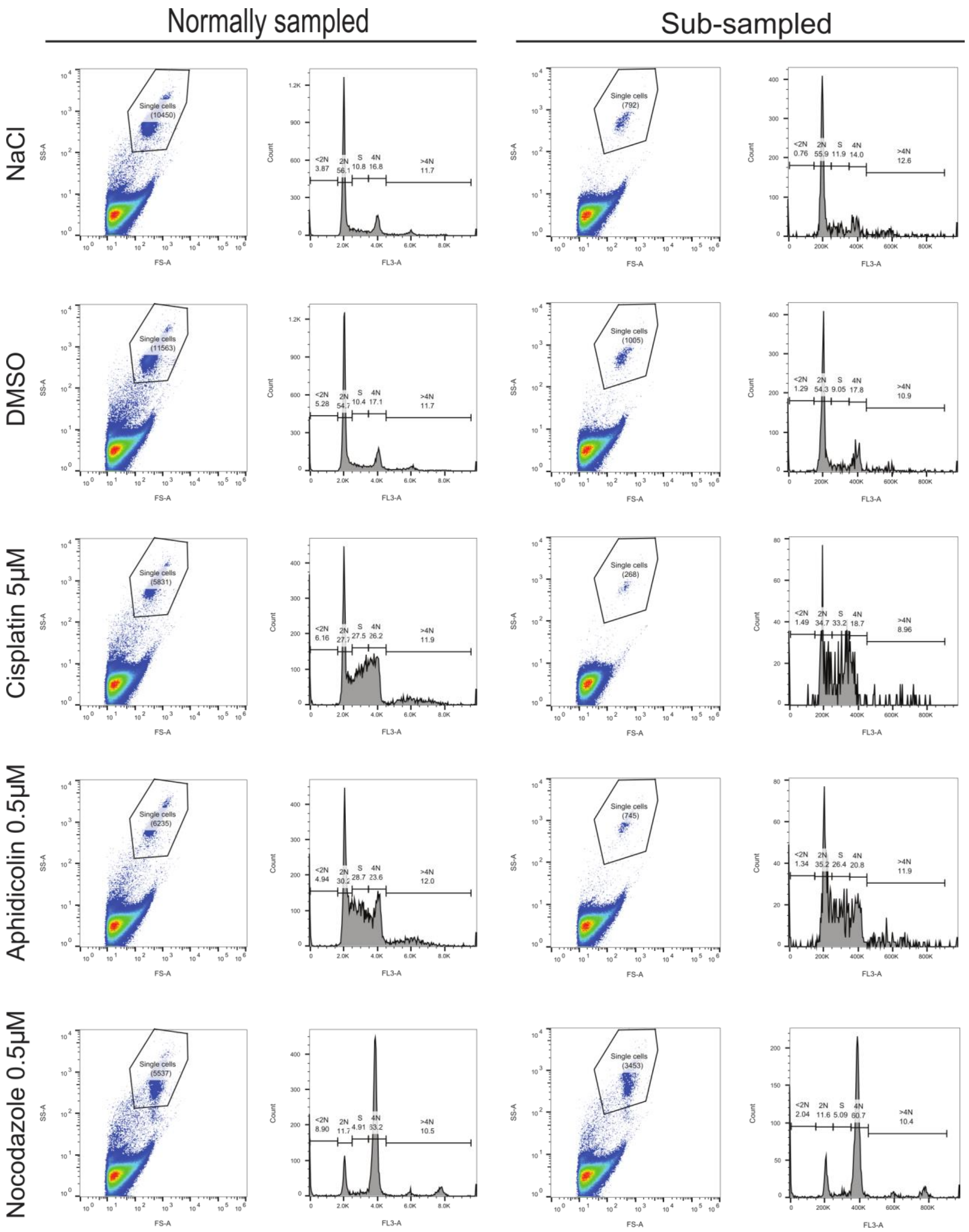


**Figure F.** Sub-sampling of the flow cytometry data. 150000 records per sample were randomly selected from the original data (with approximately 300000 records per sample). The scatter plots present the side scatter (SS) and forward scatter (FS) for each record captured with the instrument. The black polygon corresponds to manual cell selection for analysis; the number inside is the cell count. The corresponding histogram is presented to the right of each scatter plot.

## 3 APPLICATION - EDU ANALYSIS

In addition to the cell cycle analysis from imaging samples, PopulationProfiler can also be used to analyse for instance the intensity levels of other cellular staining such as EdU (5-Ehtenyl-2'-deoxyuridine), which is pyrimidine deoxynucleoside analogue that it is incorporated into the DNA during replication. When the cell's DNA replication is affected, either by the addition of a drug or by the depletion of a certain gene, the incorporation of EdU will reflect such effect by a decrease in both intensity as well as number of cells with a positive stain. This can be observed in the second row in Fig. G. Therefore, PopulationProfiler can be used as a fast tool to assess replication alterations in a high-throughput manner using EdU staining.

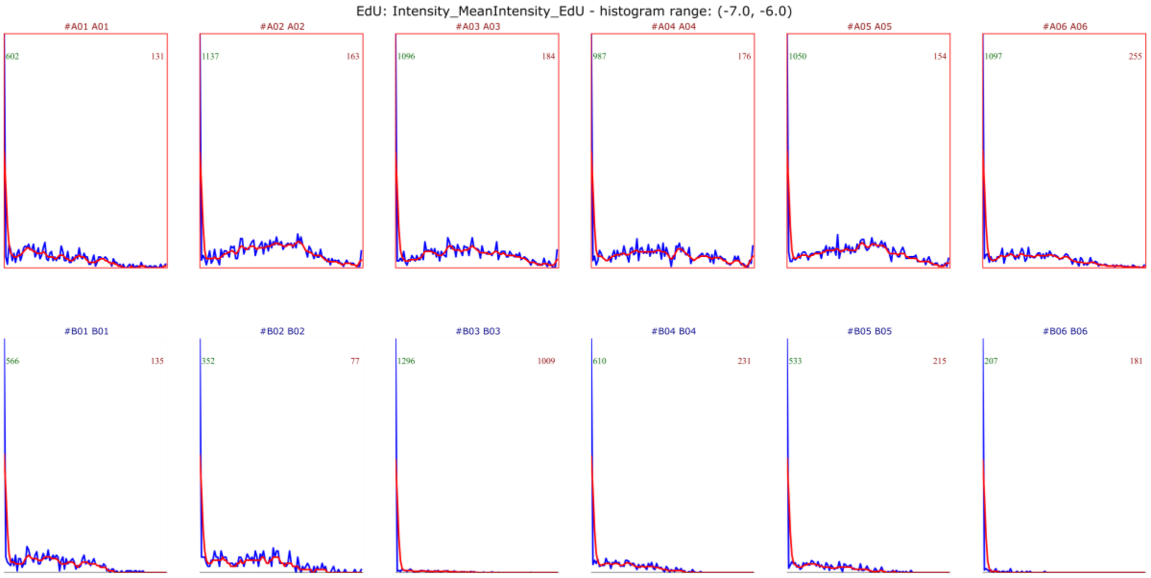


**Figure G.** Analysis of EdU incorporation.

# 4 USER MANUAL

This manual is for PopulationProfiler ver. 1.2.

## 4.1 Graphical User Interface

PopulationProfiler has a simple graphical interface (see Fig. H) that allows for selection of the input files, adjusting histogram parameters and choosing the output directory. It has a dedicated module for automatic cell cycle analysis based on DNA content but also allows manual gating selection (see Fig. I) which finds wide range of applications. The best practice is to fill the fields from top to bottom following the tips visible directly in the window or appearing after placing the mouse cursor on a given field for a couple of seconds. The interface was designed using freely available PyQt[1] library that is platform independent.

## 4.2 Input description

PopulationProfiler takes as input comma separated value (CSV) files, a commonly used format for storing datasets. The files must fulfill the following criteria:

- The first row of the file must contain names of the columns in the dataset,
- All strings in the file that contain white spaces must be quoted,
- The column with well names must have “CXX” format, where “C” is a capital character indicating the row and “XX” is a two-digit number (padded with zero if less than 10) indicating the column in the screening plate,
- More than one file can be loaded to the software, in which case each file is treated as a separate screening plate and must contain the same columns as the ones selected for the last added file (treatment label, well names and analyzed feature).

If the manual gating analysis is selected the pooled negative control histogram is displayed to aid in the selection of gates (see Fig. I). The same thresholds are later applied to all analyzed samples.

## 4.3 Output description

The primary output from the PopulationProfiler is the CSV file with the histogram data calculated for each well. This data is also visualized and stored in PDF and PNG formats (see Fig. J).

In case of the DNA content-based cell cycle analysis an additional output CSV file containing the counts and percentages of cells in each of the five cell cycle subpopulations is generated. Moreover, the total number of cells in each well and the DNA content values corresponding to 2N and 4N peaks are stored. This additional data is visualized in several ways:

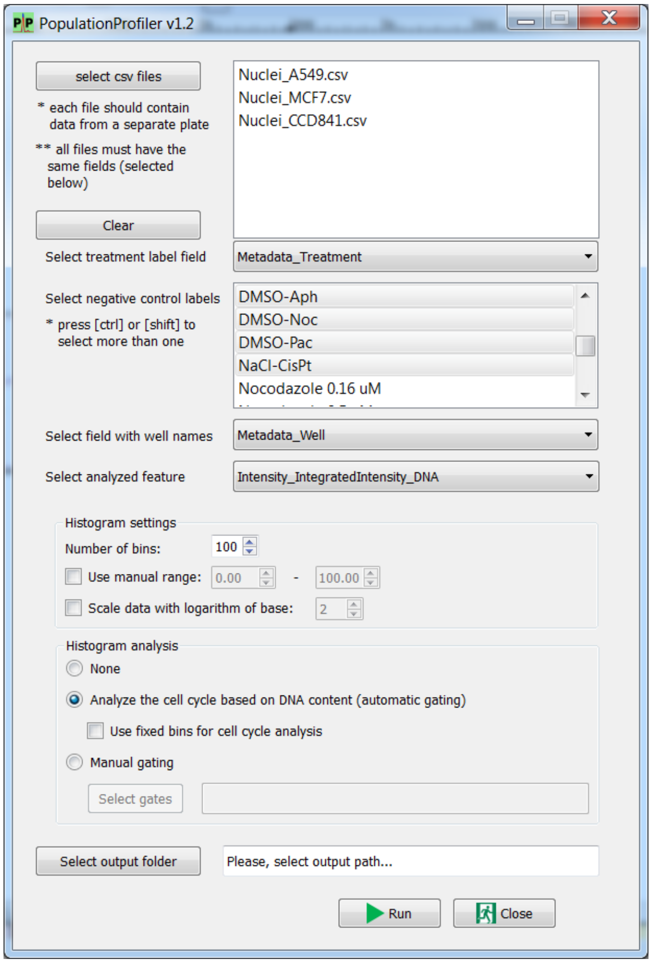


**Figure H.** Graphical user interface of the PopulationProfiler.

- Vertical black lines dividing the histogram into the cell cycle subgroups and percentage of cells in each of them are added to the graphs (see Fig. J),
- An additional stack-bar chart (see Fig. K) is generated for each input file; it presents normalized percentage contribution of each of the cell cycle subpopulations to the total cell count in the wells,
- An additional scatter plot (one per input file, not shown) presents the dependency between cell counts in the 2N and 4N subgroups. This can be particularly useful in the initial search for potentially interesting drug-dose combinations.

In case of the manual gating analysis the output is similar to that from the cell cycle analysis, except that the number of gates depends on the user selection and there is no scatter plot generated (only the histograms and stacked bar plots).

[1] http://www.riverbankcomputing.com/software/pyqt/intro

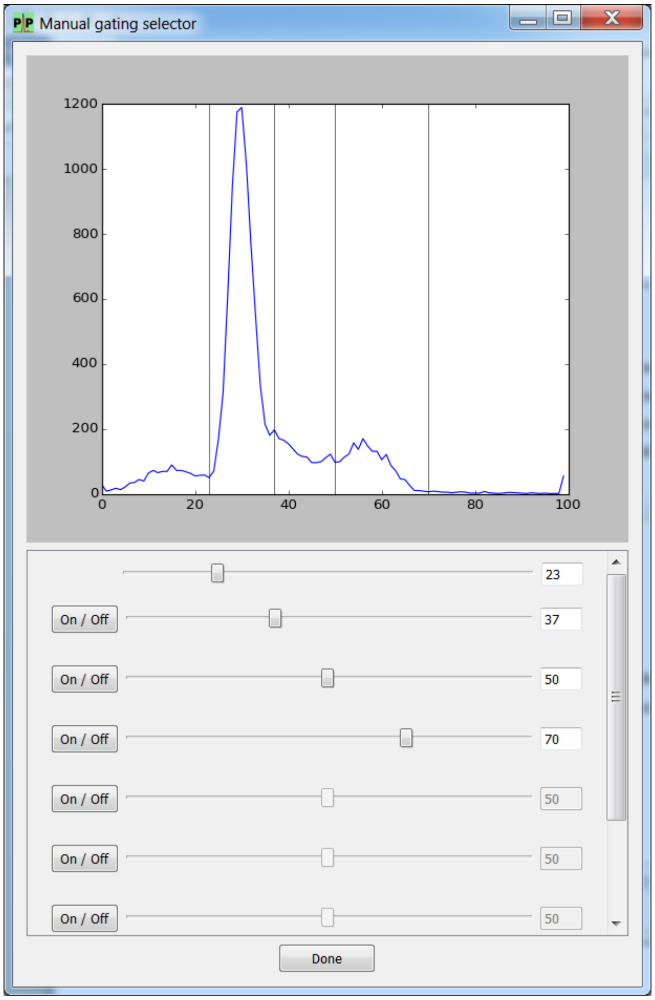


**Figure I.** Graphical user interface of the manual gating selection module in PopulationProfiler.

## REFERENCES


Carpenter, A. E. *et al.* (2006) CellProfiler: image analysis software for identifying and quantifying cell phenotypes. Genome Biology, **7**.

Chan,G.K.Y. *et al.* (2013) A simple high-content cell cycle assay reveals frequent discrepancies between cell number and ATP and MTS proliferation assays. PLoS ONE, **8**.

Ljosa, V., Sokolnicki, K. L., Carpenter, A. E. (2012) Annotated high-throughput microscopy image sets for validation. Nature Methods. **9**.

Padmanabhan, K., Eddy, W. F., Crowley, J. C. (2010) A novel algorithm for optimal image thresholding of biological data. Journal of Neuroscience Methods. **193**. 380-384.

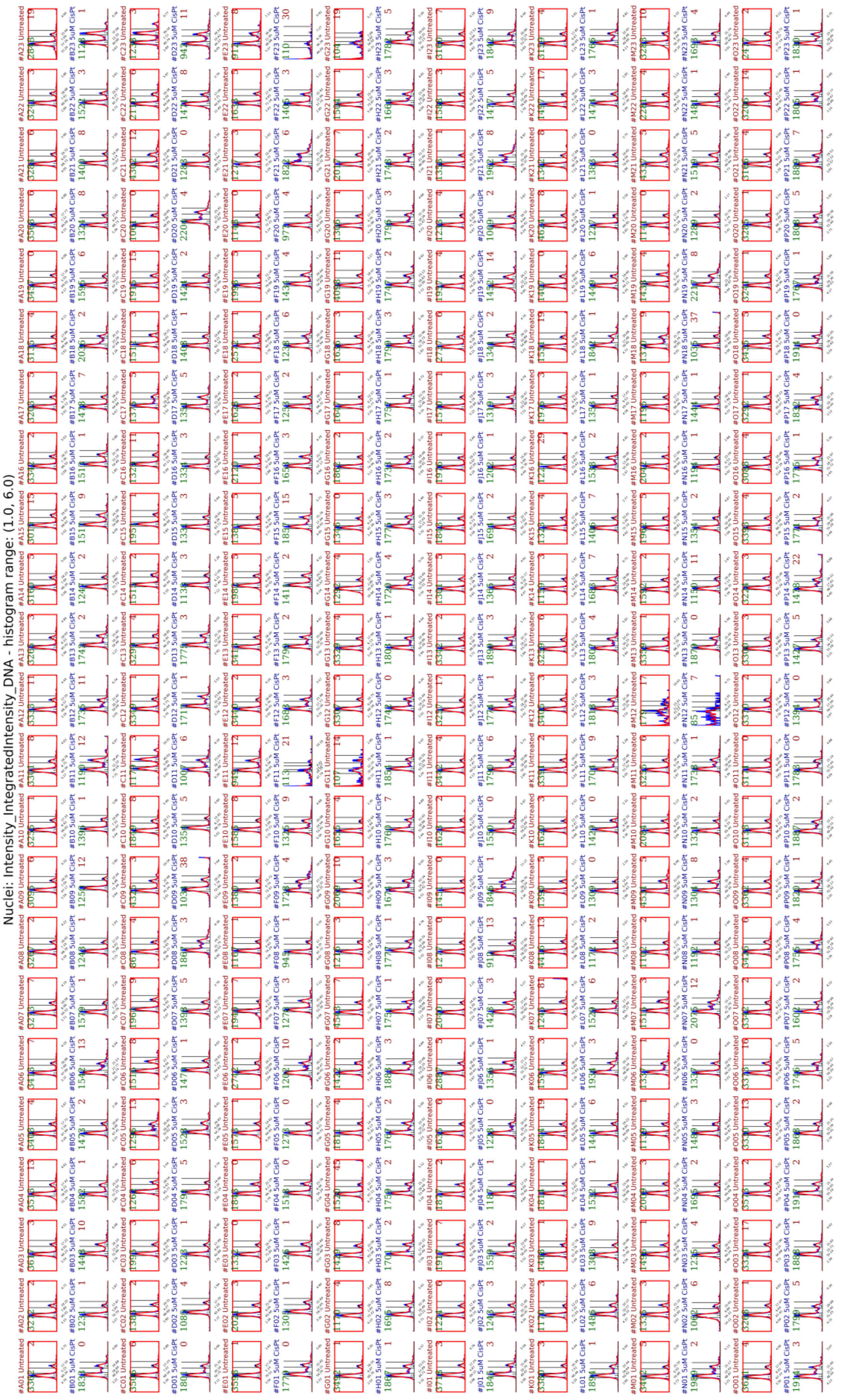


**Figure J.** Visualization of a 384-well screening plate – DNA content-based cell cycle histograms.

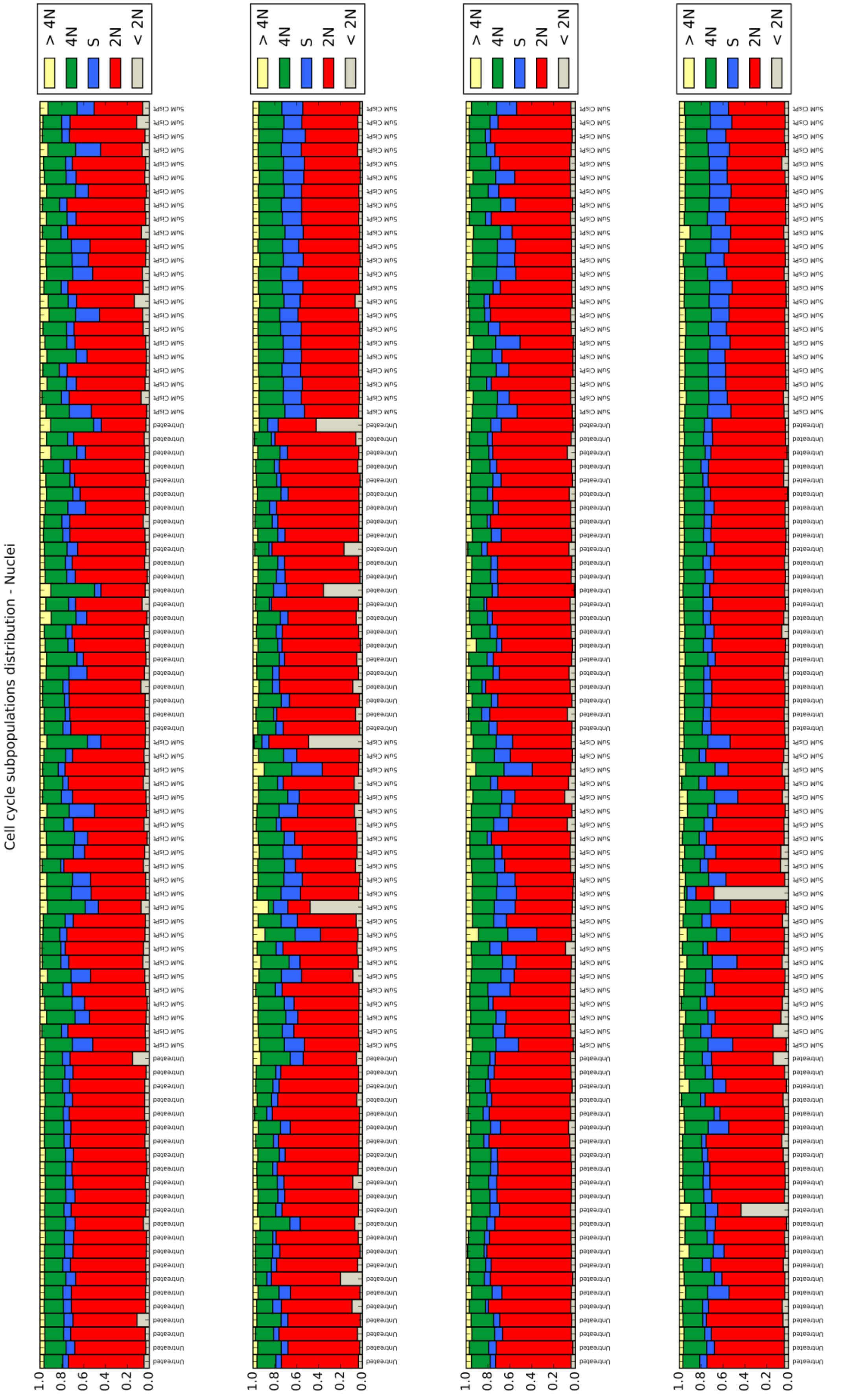


**Figure K.** Visualization of a 384-well screening plate – stack bar plots of cell cycle subpopulations based on DNA content.
